# Metal Ion Reduction, Chelation, and Cytotoxicity of Selected Bicyclic Monoterpenes and Their Binary Mixtures

**DOI:** 10.3390/metabo15030199

**Published:** 2025-03-13

**Authors:** Karolina Wojtunik-Kulesza, Marcela Dubiel, Katarzyna Klimek

**Affiliations:** 1 Department of Inorganic Chemistry, Medical University of Lublin, Chodzki Street 4a, 20-093 Lublin, Poland; marceladubiel@o2.pl; 2 Chair and Department of Biochemistry and Biotechnology, Medical University of Lublin, 1 Chodzki Street 1, 20-093 Lublin, Poland; katarzyna.klimek@umlub.pl

**Keywords:** bicyclic monoterpenes, cytotoxicity, hepatocytes, fibroblasts, metal ion reduction, chelation, synergism, FRAP, CUPRAC

## Abstract

**Background/Objectives**: Bicyclic monoterpenes are one of the most common groups of secondary plant metabolites found in Nature. Their wide spectrum of biological activity can be used in the prevention and in the treatment of various diseases, including so-called ‘diseases of civilization’. Their potential for synergistic interactions may influence the biological activities of more complex mixtures. **Methods**: This study investigated the ability of selected bicyclic monoterpenes and their binary mixtures to reduce Fe(III) and Cu(II) and chelate Fe(II) and assessed their cytotoxic activity against BJ and HepG2 cell lines. **Results**: The obtained results did not reveal synergistic interactions towards the biological activities, but binary mixtures proved to be safe in relation to the tested cell lines. Among the tested single monoterpenes, the most effective were 3-carene and *β*-pinene, with the latter exhibiting the greatest ability to decrease cell viability (CC_50_ for BJ and HepG2 cells was about 1.08 and 1.85 mM, respectively). **Conclusions**: The results revealed that both single compounds and binary mixtures demonstrate the ability to reduce selected metal ions and chelate Fe(II) ions. Synergistic interactions were not observed, but an increase in the activity of selected binary mixtures was recorded. Based on cell culture experiments, the monoterpenes and their binary mixtures can be considered safe at a concentration lower than 1 mM and close to 0.313 mM, respectively.

## 1. Introduction

The wisdom in Hippocrates’ saying: ‘Nature itself is the best physician’, is appropriate, as herbal medicine forms the foundation of medical practices in many traditions. Although modern medicine focuses on synthetic drugs, in many cases, natural compounds are the basis of them, constituting modified molecular scaffolds. Among the natural compounds, terpenes are the largest group of secondary plant metabolites in use. Long-term studies have revealed various biological activities in all of the terpene’s subgroups, including monoterpenes. However, the most important properties for human health are antioxidant/free radical scavenging, calming, antibacterial, and enzyme inhibition [1]. Natural compounds can be found in common plants used in everyday life or that surround us [2].

Many of the monoterpenes are widely used in traditional medicine. A good example is myrtenol, which is applied against microbial and bacterial infections [3] and demonstrates apoptotic activity in cancer cells [4]. Another monoterpene-myrtenal—also attracts attention because of its anti-cancer, anti-neurodegenerative, and anti-diabetic properties [5]. Among the monoterpenes, more and more attention is paid to their bicyclic compounds, which are represented by *α*- and *β*-pinene, camphor, borneol, and verbenol. These compounds, as well as the others belonging to this group, are characterized by diversified biological activity, but some also reveal cytotoxic effects (i.e., camphor) [6].

It is worth mentioning that essential oils and extracts, being mixtures of many secondary plant metabolites, show high pro-health properties. Researchers also underline the significance of these compounds’ interactions (synergism/antagonism), which can have important influences on the biological activities of the mixtures. To date, a limited number of papers have been submitted with regard to research on these impacts. An interesting example is the paper presented by Ciesla et al. [7], in which it was revealed that the activity of (±)-citronellal is increased in the presence of hydrocarbons possessing the aromatic nucleophilic ring, which may react with electrophiles. In addition to the influence of the interactions of the components of complex mixtures on their biological activity, the question of their influence on toxicity is also extremely important.

It is well-known that metal ions are crucial for the proper functioning of the human organism, but simultaneously disturbances in their homeostasis are significant in neurodegeneration development. A disturbed iron metabolism has been shown to activate oxidation and reduction reactions, leading to the formation of highly damaging hydroxyl radicals that negatively affect nerve cell function [8]. In Alzheimer’s disease (AD), increased levels of copper are observed in brain structures and, consequently, enhanced synthesis of free radicals, the inactivation of which is beyond the capacity of the affected organism. This element forms stable complexes with *β*-amyloid, which can participate in the Fenton and Haber-Weiss reactions, leading to the release of highly toxic hydroxyl radicals [9].

Despite the wide-spread presence of the monoterpenes in Nature, these compounds have not been thoroughly analyzed for their interactions and their effects on antioxidant activity. Delving into the available data, it is not difficult to find papers focused on various groups of secondary plant metabolites, while a detailed analysis of the activity of bicyclic monoterpenes appears to be incomplete. Of course, there are positions describing these activities, but upon comparing them to the extremely well-studied group of polyphenols, there is still much to be done. Furthermore, it is extremely important to analyze the activity of binary mixtures, as well as the effect of the combinations on cytotoxicity. Indeed, sometimes it appears that, despite the synergism and the significant improvement in biological activity, the combination analyzed cannot be used due to a significant increase in toxicity. Insights from synergistic interactions can guide the development of novel formulations or drugs that combine monoterpenes for optimal efficacy. This approach can inspire the synthesis of new derivatives or analogs with improved pharmacological profiles. It is highly desirable that the combination of compounds show better biological activity and at the same time do not show greater cytotoxicity than the single compounds.

**Figure 1:**
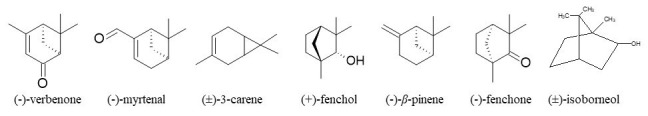
Structures of selected bicyclic monoterpenes being basis of the presented study results.

The presented paper is based on an analysis of biological activities (Fe(III) and Cu(II) reduction, Fe(II) chelation) and cytotoxic evaluation, using cell lines (BJ, HepG2) of the selected bicyclic monoterpenes: verbenone, myrtenal, 3-carene, fenchyl alcohol, *β*-pinene, fenchone, and isoborneol (Figure 1), as well as their binary mixtures. Antioxidant assays such as FRAP and CUPRAC can reveal their electron-donating capacity, helping reduce oxidative stress linked to chronic diseases. Chelation assay evaluates their metal ion-binding ability, preventing metal-induced oxidative damage. Binary mixtures of these monoterpenes may show synergistic effects, enhancing bioactivity. These findings highlight their promise in pharmaceuticals, nutraceuticals, and cosmeceuticals.

## 2. Materials and Methods

### 2.1. Materials

The bicyclic monoterpenes: (1S)-(-)-verbenone (94%), (1R)-(-)-myrtenal (95%), 3-carene (90%, mix of isomers), (1R)-(+)-fenchyl alcohol (96%), (1S)-(-)-*β*-pinene (95%), (1R)-(-)-fenchone (98%), and (±)-isoborneol (95%), as well as some reagents: TPTZ (2.4.6-Tri(2-pyridyl)-s-triazine)), ferrozine (3-(2-Pyridyl)-5,6-diphenyl-1,2,4-triazine-p,p′-disulfonic acid monosodium salt hydrate), neocuproine, trolox ((±)-6-hydroxy-2.5.7.8-tetramethylchromane-2-carboxylic acid, 97%), gallic acid (>98%), penicillin-streptomycin solution, Thiazolyl Blue Tetrazolium Bromide, and Live/Dead Cell Double Staining Kit were obtained from Merck SA (Darmstadt, Germany). Acetic acid (ACS), FeCl_3_ × 6H_2_O, FeSO_4_ × 7H_2_O, phosphoric acid (ACS), hydrochloric acid (ACS), and methanol (analytic purity grade) were obtained from Polish Reagents (Gliwice, Poland). Fetal bovine serum (FBS) was supplied by Pan-Biotech, Germany, while normal human fibroblasts (BJ cell line, CRL-2522), human hepatocellular carcinoma cells (HepG2 cell line, HB-8065), and Eagle’s Minimum Essential Medium (EMEM) were supplied by American Type Culture Collection (ATCC), London, UK.

### 2.2. Methodology

#### 2.2.1. Ferric Reducing Antioxidant Power—FRAP Method

This assay was based on a protocol we presented previously [10], but with minor modifications. The FRAP solution was prepared prior to analysis by mixing (10:1:1 *v/v/v*) 0.3 M acetate buffer (pH 3.6), 0.01 M TPTZ in 0.04 M HCl, and 0.02 M FeCl_3_ × 6H_2_O (kept in the dark). The analysis was performed using a 96-well plate. Eight concentrations of the monoterpenes were studied: from 5 mM to 0.039 mM—obtained by two-fold serial dilutions. Thereafter, 30 s before the first scan, there was an orbital shaking at 400 rpm. Absorbance was measured at 593 nm using a SPECTROstar Nano microplate reader while maintaining a temperature of 37 °C throughout the measurement. The total length of analysis of a single plate was 60 min and consisted of 13 cycles of 5 min. A FRAP working solution with methanol instead of a sample was used as a blank. The results were calculated into μg/mL Fe^2+^ on a calibration curve that was prepared analogically using an aqueous solution of FeSO_4_ at concentrations of 0.5–2.5 μg/mL (y = 0.9406x − 0.0115, R^2^ = 0.9964), as well as Trolox equivalents based on a calibration curve prepared for concentrations of 0.2–3.0 μg/mL (y = 0.7016x − 0.0009, R^2^ = 0.9983). All measurements were carried out in triplicate.

#### 2.2.2. FRAP—Synergism Studies

The studies performed towards the synergism of selected monoterpenes were prepared analogically according to a previously presented method with slight modification. Synergism was tested in the concentration range of 5 mM–0.039 mM against each of the terpenes in the mixture. The mixture was incubated for 15 min in order to allow time for possible interactions. The remainder of the study was carried out as described in the FRAP methodology for single compounds.

#### 2.2.3. Fe(II) Chelation Assay—Ferrozine Based Study

The assay was performed based on a previously described procedure [11], but with slight modification. Eight concentrations of the monoterpenes were studied: from 5 mM to 0.039 mM as obtained by two-fold serial dilutions. The analyzed samples of monoterpenes were mixed with FeSO_4_ solution (2 mM) and incubated for 10 min in the dark. Subsequently, a ferrozine solution (5 mM) was added, and the solution was re-incubated for another 10 min in the dark. Afterwards, MeOH was added, and absorbance was measured at 562 nm. Prior to the actual measurements, the plate was shaken orbitally inside the reader at 400 rpm for 30 s. The wells to be analyzed were orbital scanned within a 4 mm radius for a period of 20 min. EDTA, a strong metal ion chelator, was used as a reference compound. EDTA equivalents were calculated based on a calibration curve (y = −2.7265x + 4.1016, R^2^ = 0.9973).

#### 2.2.4. Fe(II) Chelation Assay—Synergism Studies

The synergism studies were performed analogously to a Fe(II) chelation assay performed for single compounds. Synergism was tested in the concentration range of 5 mM–0.039 mM against each of the terpenes in the mixture. The remainder of the study was carried out as described in the ‘Fe(II) chelation assay–ferrozine based study’ Section 2.2.3.

#### 2.2.5. Cu(II) Reduction—CUPRAC Assay

The assay was performed in accordance with the methodology presented in our earlier work [11], but with slight modification. Similarly to previous studies, eight concentrations of the monoterpenes were studied: from 5 mM to 0.039 mM as obtained by two-fold serial dilutions. The solutions of CuCl_2_ (0.01 M), ammonium buffer (1 M. PH = 7), and neocuproine (0.0075 M) were prepared daily. These were added to the monoterpenes previously applied onto the plate and incubated for 15 min in the dark at room temp. Following this procedure, absorbance was measured at 450 nm with a reader that scanned the wells orbitally within a 4 mm radius for 20 min. Prior to the actual analysis, orbital shaking occurred at 400 rpm for 30 s.

#### 2.2.6. CUPRAC—Synergism Assay

In this case, the monoterpenes were mixed analogousy to the FRAP and chelation assays. The microplate with the binary mixtures was incubated at room temperature for 10 min. The appropriate reagents were then added, and re-incubation occurred for 15 min. Absorbance was subsequently measured. In both CUPRAC assays, trolox was used as a standard reagent, and a calibration curve was prepared (y = 1.212x + 0.742, R^2^ = 0.995).

#### 2.2.7. Cytotoxicity Evaluation

The cytotoxic activity of single monoterpenes, as well as their combinations, was evaluated towards two cell lines: normal human skin fibroblasts (BJ cell lines, CRL-2522, ATCC, USA) and human hepatocellular carcinoma cells (HepG2 cell line, HB-80-65, ATCC, USA). The fibroblasts (BJ cells) were selected for this study because these cells are considered to be model cells for assessing the cytotoxicity of various compounds with biomedical potential [12]. In turn, the hepatocytes (HepG2 cells) are often used for evaluation of compounds due to the involvement of liver cells in drug metabolism [13]. Cells were cultured according to the supplier’s (ATCC) guidelines, i.e., they were maintained in Eagle’s Minimum Essential Medium (EMEM, 30-2003, ATCC), 37 °C, 5% CO_2_, and 95% humidity. In the first stage of the experiment, the cytotoxic activity of single monoterpenes was assessed. For this purpose, serial dilutions of the compounds in the concentration range of 5–0.0097 mM were prepared in culture medium, and then they were added to cell cultures–BJ cells were seeded at concentrations of 2 × 10^4^ cells/well, while HepG2 cells were seeded at concentrations of 3 × 10^4^ cells/well. After 24 h of incubation, cell viability was evaluated via MTT assay. Next, CC_50_ values, i.e., concentrations that inhibit cell viability by 50% compared to control, were determined using 4-parameter nonlinear regression analyses. GraphPad Prism 5, version 5.04, was employed for this purpose. In the next step, the cytotoxicity of the mixtures of compounds was assessed (combinations with potential synergistic activity were selected based on previous experiments and are listed in Table 2 in Section 3.1—FRAP assay). For this purpose, monoterpenes at non-toxic concentrations (0.312 mM) were combined together, added to the BJ and HepG2 cells, and incubated for 24 h. The MTT assay was then performed in order to establish cell viability. The results are presented as a percentage of cell viability in relation to control cells, i.e., incubated without tested compounds (viability considered as 100%). The cells were subsequently stained utilizing a Live/Dead Double Staining Kit for qualitative assessment of cell viability. For this purpose, cells were observed under a confocal laser scanning microscope (CLSM, Olympus Fluoview equipped with FV1000, Japan).

**Figure 2:**
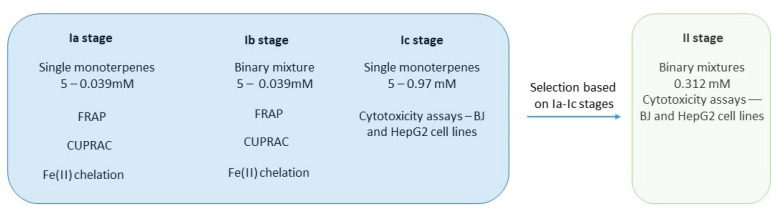
Schematic diagram of performed studies.

All of the performed assays are presented as a schematic diagram (Figure 2).

#### 2.2.8. Statistical Analysis

Statistical analysis was conducted using an internal program written in MARS. SPECTROstar Nano wafer reader control software. Cell culture experiments were performed as three independent replications, and the results were presented as mean values ± standard deviations (SD). Obtained data were analyzed using GraphPad Prism 5 Software, Version 5.04, so as to prepare graphs, to evaluate the normal distribution of results, as well as to determine statistical differences (D’Agostino and Pearson omnibus normality test followed by unpaired Student *t*-test; differences with *p* < 0.05 were marked as statistically significant).

## 3. Results

### 3.1. FRAP Assay

**Figure 3:**
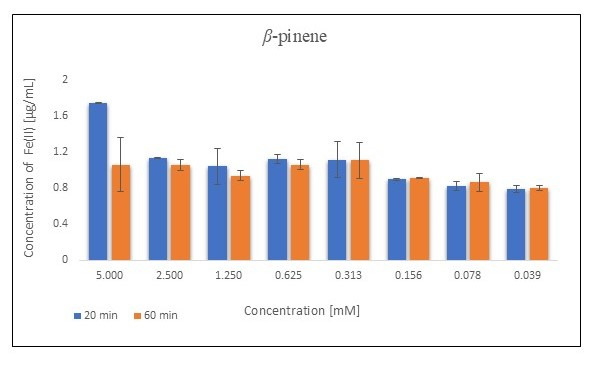
FRAP assay results obtained for β-pinene presented in the form of concentration of Fe(II) [µg/mL]. (n = 4; mean values ± SD).

Among the bicyclic monoterpenes tested, the highest activity was observed for 3-carene, *β*-pinene, and verbenone, as indicated by slight variations in absorbance with time. 3-Carene, despite good activity, was characterized by poor stability with time. *β*-pinene, on the other hand, was characterized by high activity at the beginning of the measurements (at 20 min) (Figure 3). However, its stability deteriorated with time, as indicated by the absorbance results obtained at 60 min of plate scanning.

**Figure 4:**
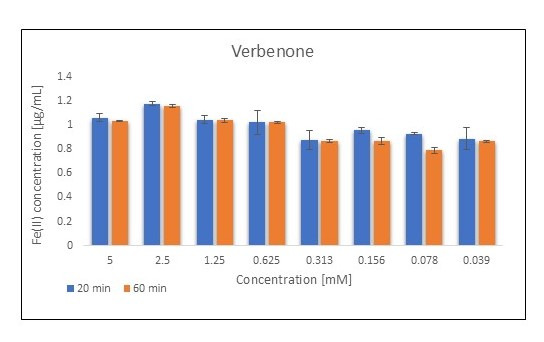
FRAP assay results obtained for verbenone at various concentrations (n = 4; mean values ± SD).

Another bicyclic monoterpene that showed a reducing potential for Fe(III) ions is verbenone. This compound is characterized by a small change in absorbance between 20 and 60 min of the absorbance value reading and can be considered stable under the experimental conditions. The significant advantage of the monoterpene is the stability of the Fe(III) reduction, as well as showing comparable activity for all used concentrations (Figure 4).

The obtained absorbance values for the tested terpenes were converted into trolox equivalents and Fe(II) ion concentrations [μg/mL] as obtained from the reduction in Fe(III) ions by the tested terpenes. The results for 5 mM and 0.312 mM are presented in a summary table (Table 1). From the data obtained, it can be concluded that better values were obtained for β-pinene, which showed both a high trolox equivalent value and Fe(II) ion concentration. It is noteworthy that in the case of β-pinene, the activity per the aforementioned quantities decreased significantly 60 min after the initiation of the reaction. Verbenone and isoborneol also showed satisfactory activity.

The next step in the study was to determine the reduction capacity of Fe(III) ions by mixing two selected monoterpenes in single wells of the plate. Synergism studies were performed for selected concentrations, but the best activity was observed for a concentration of 0.312 mM in relation to each of the compounds in a two-component combination. The outcome of this endeavor displayed the highest recorded increase in activity, as compared to the activity of single compounds. The obtained studies, presented in the form of trolox equivalents and Fe(II) concentration, are listed in Table 2.

As can be seen from the results presented in Table 1 and Table 2, the trolox equivalents and Fe(II) concentration were higher for the binary mixtures than for the individual compounds, but none of the combinations achieved synergism, i.e., a value greater than the sum of the actions of the individual terpenes.

### 3.2. Fe(II) Chelation Assay

**Figure 5:**
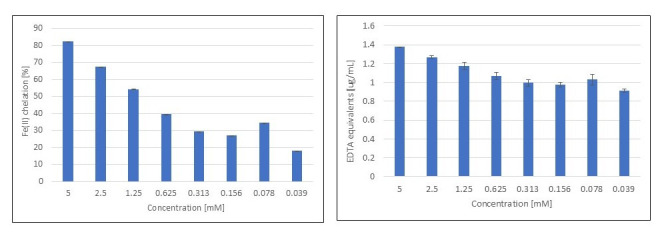
EDTA equivalents [µg/mL] and Fe(II) chelation [%] obtained for *β*-pinene using ferrozine-based coloring method (*n* = 4, mean values ± SD).

The activity of selected compounds towards the chelation of Fe(II) ions was determined by applying the ferrozine colorimetric method. The results obtained showed varying metal ion binding capacities with regard to the tested monoterpenes. The determined chelating activity (%) was highest for β-pinene and reached values > 50% at higher concentrations (Figure 5). Another monoterpene that showed Fe(II) ion binding capacities of >70% at lower and higher concentrations was 3-carene. The remaining compounds showed lower chelating activity, especially at lower concentrations. Monoterpenes such as fenchyl alcohol, fenchone, myrtenal, and verbenone showed a very good Fe(II) ion chelating ability (of >80%) only when higher concentrations were used (Table 3).

Studies performed to determine possible synergism between monoterpenes in a binary mixture did not reveal any interactions. The values obtained for the most active mixtures were, however, slightly higher than the results obtained for a single monoterpene, having a higher chelating potential for Fe(II) ions.

### 3.3. CUPRAC Assay

We assessed the ability of selected bicyclic monoterpenes to reduce Cu(II) ions by applying the CUPRAC (Cupric Ion Reducing Antioxidant Capacity) colorimetric method. The reference compound in the method performed was trolox. Based on the absorbance values obtained for this compound, a standard curve was drawn up.

**Figure 6:**
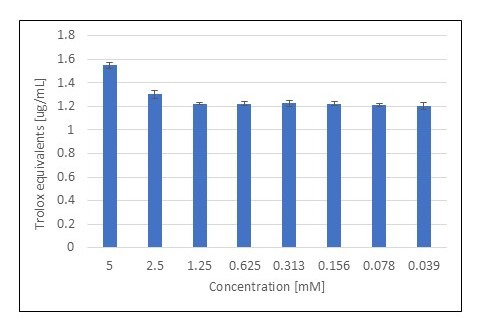
The ability of 3-carene to Cu(II) reduction is based on the CUPRAC assay (n = 4, mean values ± SD).

Accordingly, with regard to Trolox equivalents, the most active monoterpene towards Cu(II) reduction turned out to be 3-carene (Figure 6).

Among the other compounds, β-pinene showed high absorbance values (0.661 ≤ A ≤ 1.685), especially at higher concentrations, indicating a moderate reducing potential for Cu(II) ions as compared to the most active monoterpenes. In contrast, compounds such as fenchyl alcohol, isoborneol, fenchone, myrtenal, and verbenone showed low activity in the CUPRAC assay. Their absorbance values at all concentrations tested were significantly lower compared to 3-carene, indicating their low copper ion reducing potential. Trolox equivalents for selected concentrations of monoterpenes are presented in Table 4.

Similarly to previous assays, the best activity was observed for a concentration of 0.312 mM in relation to each of the compounds in a two-component combination. Indeed, the highest increase in activity was recorded for this concentration as compared to single compounds. Unfortunately, there was no significant increase in Cu(II) reduction activity seen in the testing of all by binary mixtures. In the case of some mixtures, trolox equivalents were slightly higher than for single compounds, but this cannot be interpreted as a synergistic effect.

### 3.4. Cytotoxicity Evaluation

**Figure 7:**
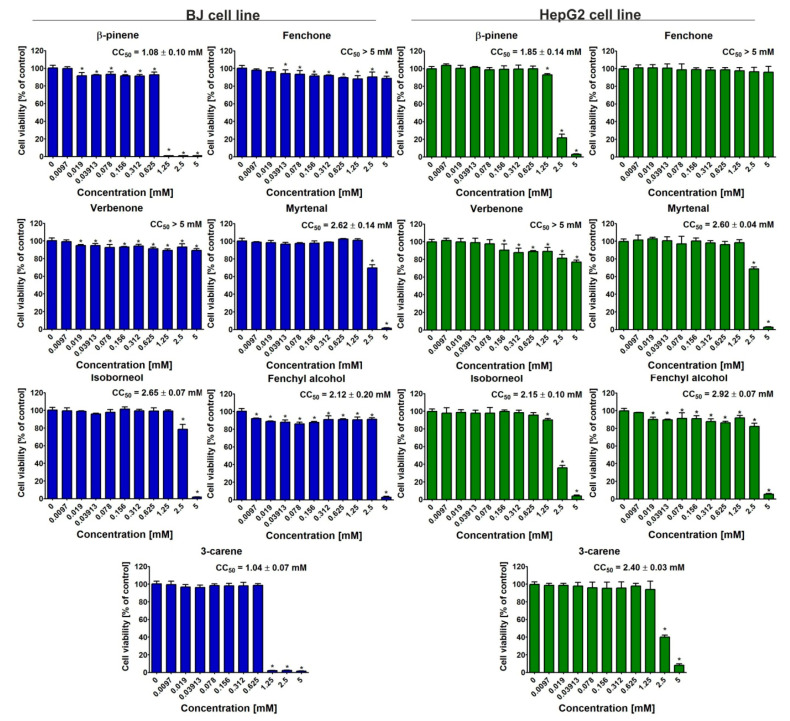
Viability of normal human fibroblasts (BJ cell line) and human heapocellular cells (HepG2 cell line) after 24 h incubation with bicyclic monoterpenes at concentrations ranging from 5 to 0.0097 mM. Metabolic activity was evaluated using the MTT assay, and results were presented as a percentage value compared to control cells (untreated, 0 mM). * Statistically significant differences compared to control cells, unpaired Student *t*-test, *p* < 0.05.

**Figure 8:**
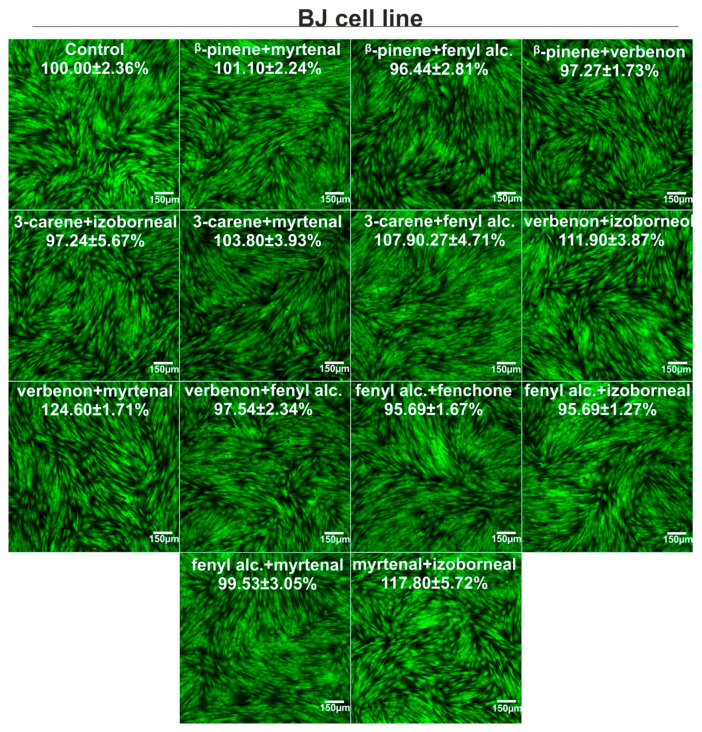
Viability of normal human fibroblasts (BJ cell line) after 24 h incubation with mixtures of bicyclic monoterpenes, which exhibited the best activity during metal ion reduction and chelation assays. The compounds were used in non-cytotoxic concentrations, i.e., 0.312 mM. Metabolic activity was evaluated using the MTT assay, and results were presented as percentage values compared to control. Confocal microscope images were taken after staining cells with the Live/Dead Double Staining Kit–live cells emitted green fluorescence, while dead cells generated red; Magnification ×100, scale bar = 150 μm.

**Figure 9:**
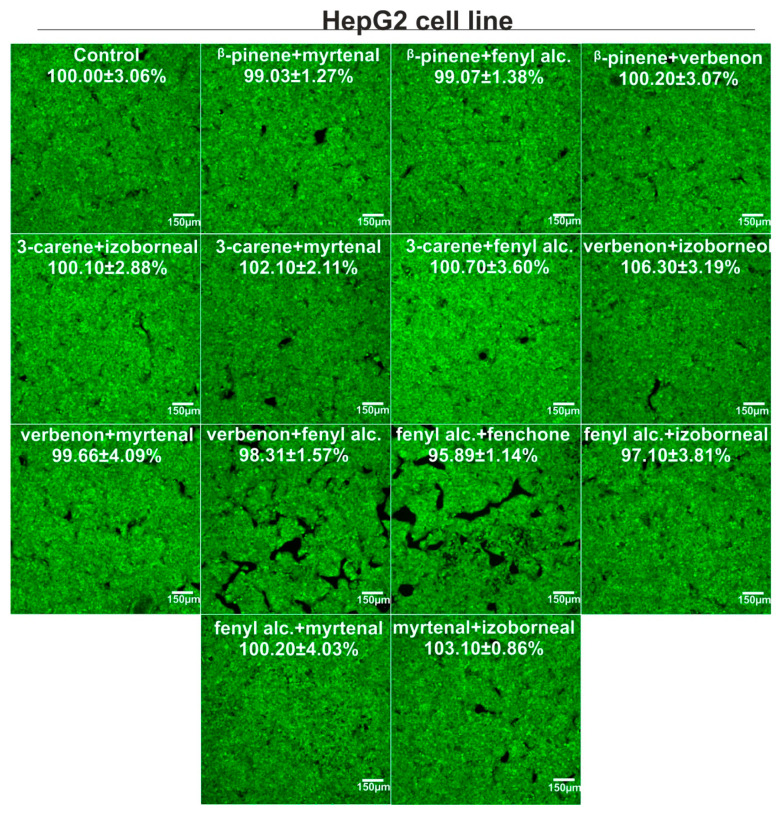
Viability of human hepatocellular carcinoma cells (HepG2 cell line) after 24 h incubation with mixtures of bicyclic monoterpenes, which exhibited the best activity during metal ion reduction and chelation assays. The compounds were used in non-cytotoxic concentrations, i.e., 0.312 mM. Metabolic activity was evaluated using the MTT assay, and results were presented as a percentage value compared to the control. Confocal microscope images were taken after staining cells with the Live/Dead Double Staining Kit–live cells emitted green fluorescence, while dead cells generated red; Magnification ×100, scale bar = 150 μm.

Bicyclic monoterpenes showed different cytotoxicity in vitro towards normal human fibroblasts (BJ cells) and human hepatocellular carcinoma cells (HepG2 cells), as shown in Figure 7. In general, β-pinene exhibited the greatest ability to decrease cell viability (CC_50_ for BJ and HepG2 cells was about 1.08 and 1.85 mM, respectively), while fenchone and verbenone decreased metabolic activity of both cell types the least (CC_50_ > 5 mM). In the case of combinations of bicyclic monoterpenes, it was noted that the mixtures did not demonstrate cytotoxic activity towards both cell types, as cell viability was above 95% compared to control cells (approx. 100%), as shown in Figure 8 and Figure 9. Interestingly, some combinations even promoted cell viability compared to control cells. Such a phenomenon was observed for the following combinations of bicyclic monoterpenes: 3-carene + myrtenal, verbenone + myrtenal, and verbenone + myrtenal (in this case only for BJ cells), which indicates their high biomedical potential under in vitro conditions.

## 4. Discussion

Bicyclic monoterpenes, which are constituents of a wide range of essential oils, exhibit many health-promoting properties, including antioxidant activity. Such activity plays an important role in the prevention and treatment of many neurodegenerative disorders, including, but not limited to, Alzheimer’s and Parkinson’s disease. This group of secondary plant metabolites has often been evaluated for neuroprotection. Studies of note in this regard included those performed by Dahiya et al. [14], who evaluated *β*-pinene activity against sporadic AD. Using a rat model, they saw neuroprotective activity of the monoterpene based on reduction in oxidative stress and mitochondrial dysfunction, which, in turn, led to improvement in cognitive functions. Other interesting results were presented by Woo et al. [15], who focused on 3-carene. They demonstrated that oral administration of 3-carene to C57BL/6N mice prolongs sleep duration and shortens sleep onset in the pentobarbital-induced sleep test. Accordingly, 3-Carene enhances GABA_A_ receptor-mediated synaptic activity by extending the decay time constant of inhibitory synaptic responses. These findings suggest that 3-carene exerts sleep-promoting effects by acting as a positive modulator of the GABA_A_-BZD receptor.

In analyzing the biological activity of secondary plant metabolites, it is important to underline the significance of their possible synergistic interactions. These can be responsible for the high activity of the metabolite mixture. As the combined action of multiple metabolites can produce stronger effects than individual compounds alone, this effect can lead to greater therapeutic benefits at lower doses. Additionally, synergism can allow for minimizing potential side effects and toxicity, which is essential for safe pharmaceutical application. Moreover, some metabolites work together to target multiple biological pathways, improving efficacy against diseases such as infections and cancer, as well as inflammation [16].

The studies presented in this paper are based on a selected group of terpenes and involved determining the ability of these compounds to chelate Fe(II), as well as to reduce Fe(III) and Cu(II) ions. Colorimetric spectrophotometric methods were applied in this endeavor. The monoterpenes showing the highest activity in the three undertaken tests were *β*-pinene and 3-carene. Terpene compounds are currently of interest as potential substances with the ability to neutralize free radicals. In analyzing the results obtained, it is possible to see some correlation between the structure of the monoterpenes studied and their antioxidant activity. Their potential to reduce iron (III) ions and thus to inhibit many adverse reactions in the body has been the subject of much research. The compound with the weakest reducing potential for Fe(III) and Cu(II) ions in the FRAP and CUPRAC methods was fenchone. In contrast, when the properties towards chelation of Fe(II) ions were tested, isoborneol showed the lowest activity. The combination of single terpenes in a 1:1 ratio did not yield expected results, as no synergism was obtained for the mixtures made. The most active combinations usually included one of the most active monoterpenes, i.e., *β*-pinene and 3-carene.

Excessive concentrations of selected metals, including Fe(II) iron ions, can be a direct risk factor for the development of neurodegenerative diseases. It is therefore important to find effective methods to nullify the negative effects of these compounds on the functioning of the human body. Chelation of metal ions may be important because of the ability to avoid the formation of ROS, which can induce damage to cellular structures. Most commonly, this process involves the association of a ligand molecule or ion with a central metal atom via a coordination bond. Based on various studies, the formation of a metal ion-test compound complex, in this case, bicyclic monoterpenes, is influenced by the presence of specific functional groups in the structure of the compounds. Gulcin et al. showed that curcumin, when used as a food ingredient, has a high chelating capacity for Fe(II) ions. The compound was revealed to have the ability to form complexes mediated by the presence of active -OH and -OCH_3_ groups. In addition, structures containing functional groups such as C=O and C-OH can readily bind metal ions [17].

In analyzing the structures of the bicyclic monoterpenes that demonstrated the highest chelation activity, i.e., *β*-pinene and 3-carene, it can be seen that these compounds do not possess the aforementioned functional groups, favoring the formation of complexes with Fe(II) ions. Verbenone, myrtenal, and fenchone, containing the -C=O group in their structures, showed chelation at >60% for selected concentrations. In contrast, isoborneol and fenchyl alcohol, which have an -OH group in their molecule, were characterized by a different activity, which reached a maximum activity of 13.8% at a concentration of 0.625 mM for isoborneol and 80% for fenchyl alcohol at a concentration of 50 mM.

The CUPRAC method, using electron transfer (ET), is based on the measurement of the absorbance of Cu(I)-neocuproine chelate (Nc), which is formed by the redox reaction between chain-splitting antioxidants and the CUPRAC reagent. As the concentration of antioxidants increases, the absorbance value and color intensity of the test solution also increase. Polyphenols, considered standard antioxidants, contain active hydroxyl groups (Ar-OH) in their structure, which are oxidized to quinones (Ar=O), while Cu-Nc (Neocuproin) is reduced to Cu(Nc)_2_^+^ chelate, which has an orange-yellow color. The number and position of the -OH groups, as well as the degree of coupling of the molecule, have been shown to be responsible for electron transfer. This was confirmed by studies in which the activities of fisetin and quercetin were determined. In our work, it turned out that fisetin, having fewer hydroxyl groups, achieved lower TEAC values compared to the other compound [11]. Analyzing the structure of the monoterpenes studied, it can be concluded that the structures showing the highest activity did not have -OH groups in their molecules. Only isoborneol and phenchyl alcohol, having a low reducing potential for Cu(II) ions, contained hydroxyl groups in their molecules. The activity was also unaffected by the presence of a carbonyl group (>C=O) in the molecules of such monoterpenes as verbenone, fenchone, and myrtenal. In contrast, the structures for which high absorbance values were obtained had double bonds, which are probably crucial for the activity of monoterpenes in the CUPRAC assay. As previously mentioned, in the DPPH^•^ radical assay, compounds exhibiting high antioxidant potential had π-bonds in their structure, supporting the proposed hypothesis [18].

In our work, we assessed cell viability using the MTT test and after staining live/dead cells. The MTT test is one of the most popular methods for investigating cell viability, as it allows for a quantitative assessment of their metabolic activity. So far, it has been eagerly used to determine the cytotoxic activity of single compounds, as well as their combinations (e.g., with synergistic action). In turn, staining cells with calcein AM and propidium iodide (Live/Dead Double Staining kit) allows for a qualitative assessment of cells and is also often chosen to ascertain the cytotoxicity of single compounds and/or their combinations [19,20,21]. We obtained significant results in the undertaken cytotoxic tests, performing them on BJ and HepG2 cell lines. The greatest ability to decrease cell viability was observed for *β*-pinene (CC_50_ = 1.08 mM (BJ cells) and 1.85 mM (HepG2 cells). In the case of the binary mixture, the concentration was selected based on the first stage of the experiments, namely, Fe(III) and Cu(II) reduction and Fe(II) chelation. The results revealed that a concentration of 0.312 mM turned out to be the most effective in the context of synergism interactions. The analyzed binary mixture did not, however, reveal a cytotoxic effect towards both the cell lines at the aforementioned concentration.

## 5. Conclusions

In summary, most of the tested compounds demonstrated the ability to reduce Fe(III) and Cu(II) ions and chelate Fe(II) ions, suggesting their potential role in metal-related biological processes. Among the individual monoterpenes, 3-carene and *β*-pinene were the most effective, with *β*-pinene exhibiting the greatest cytotoxic effect: CC_50_ = 1.08 mM (BJ cells) and 1.85 mM (HepG2 cells). The performed studies did not confirm synergistic interactions between the tested bicyclic monoterpenes in terms of their biological activities. While no true synergy was found, some binary mixtures did, however, show an increase in activity, suggesting potential benefits of combining certain monoterpenes. Overall, the findings highlight the potential of bicyclic monoterpenes and their mixtures in pharmaceutical and biomedical applications, particularly for their metal ion-modulating properties and safety profiles. Moreover, based on the available literature, these monoterpenes are known to exhibit promising antioxidant potential and additional properties, including anticancer or anti-inflammatory activity. However, in order to confirm their activity, a number of additional in vitro and in vivo studies should be conducted to determine their efficacy and safety in contact with the human body. In order to confirm the investigated activities, which can be used in medicine, it is necessary to carry out more detailed wide-spectrum studies. Unfortunately, the results presented in this paper do not give a complete view of the activity of the compounds studied towards metal reduction and chelation. It should be noted that the in vitro studies are the first step towards more advanced research, which will confirm or negate the activity of these compounds.

## Figures and Tables

**Table 1:** Bicyclic monoterpene study results towards Fe(III) reduction presented as Trolox equivalents [µg/mL] and Fe(II) concentration [µg/mL] (n = 4; mean values ± SD).

Monoterpene	Concentration (mM)	Trolox Equivalents [µg/mL]		Fe(II) Concentration [µg/mL]	
		20 min	60 min	20 min	60 min
*β*-Pinene	5	2.487 ± 0.016	1.507 ± 0.464	1.868 ± 0.012	1.137 ± 0.350
	0.312	1.585 ± 0.293	1.578 ± 0.324	1.196 ± 0.221	1.190 ± 0.244
Fenchone	5	1.266 ± 0.073	1.243 ± 0.060	0.957 ± 0.055	0.940 ± 0.048
	0.312	1.039 ± 0.021	1.048 ± 0.020	0.788 ± 0.016	0.795 ± 0.016
Verbenone	5	1.511 ± 0.051	1.472 ± 0.004	1.140 ± 0.038	1.112 ± 0.003
	0.312	1.249 ± 0.101	1.233 ± 0.015	0.945 ± 0.077	0.933 ± 0.011
Myrtenal	5	1.360 ± 0.009	1.336 ± 0.010	1.028 ± 0.007	1.009 ± 0.008
	0.312	1.223 ± 0.044	1.210 ± 0.058	0.925 ± 0.033	0.916 ± 0.044
Isoborneol	5	1.236 ± 0.066	1.227 ± 0.084	0.935 ± 0.050	0.929 ± 0.063
	0.312	1.193 ± 0.109	1.165 ± 0.085	0.903 ± 0.082	0.882 ± 0.064
Fenchyl alcohol	5	1.274 ± 0.034	1.257 ± 0.050	0.964 ± 0.026	0.951 ± 0.038
	0.312	1.357 ± 0.013	1.319 ± 0.012	1.025 ± 0.010	0.997 ± 0.009
3-Carene	5	1.926 ± 0.036	1.525 ± 0.131	1.450 ± 0.027	1.151 ± 0.099
	0.312	0.954 ± 0.039	0.894 ± 0.037	0.725 ± 0.030	0.680 ± 0.028

**Table 2:** Trolox equivalents (gray boxes) and Fe(II) concentrations (yellow boxes) obtained for the bicyclic monoterpene combinations tested at a concentration of 0.312 mM at 20 and 60 min of scanning (lighter and darker colors, respectively), in the FRAP assay (n = 4, mean values ± SD).

	*β*-pinene	fenchone	3-carene	verbenone	myrtenal	isoborneol	fenchyl alcohol
*β*-pinene	x	1.075 ± 0.038	1.072 ± 0.057	1.048 ± 0.048	1.070 ± 0.064	1.085 ± 0.082	1.050 ± 0.104
		1.124 ± 0.032	1.059 ± 0.051	1.032 ± 0.036	1.059 ± 0.073	1.039 ± 0.045	1.055 ± 0.106
fenchone	1.457 ± 0.051	x	0.954 ± 0.048	1.075 ± 0.122	0.917 ± 0.019	0.966 ± 0.039	0.971 ± 0.032
	1.522 ± 0.043		0.933 ± 0.049	1.059 ± 0.122	0.906 ± 0.022	0.941 ± 0.042	0.951 ± 0.019
3-carene	1.453 ± 0.078	1.294 ± 0.065	x	0.728 ± 0.012	0.896 ± 0.084	0.902 ± 0.033	0.995 ± 0.106
	1.435 ± 0.069	1.266 ± 0.066		0.723 ± 0.010	0.879 ± 0.090	0.878 ± 0.022	0.968 ± 0.094
verbenone	1.420 ± 0.065	1.457 ± 0.166	0.991 ± 0.017	x	0.832 ± 0.039	0.792 ± 0.069	1.031 ± 0.059
	1.398 ± 0.049	1.435 ± 0.166	0.985 ± 0.013		0.802 ± 0.036	0.800 ± 0.064	1.013 ± 0.072
myrtenal	1.450 ± 0.087	1.244 ± 0.026	1.216 ± 0.114	1.130 ± 0.053	x	0.887 ± 0.037	0.917 ± 0.023
	1.435 ± 0.099	1.230 ± 0.030	1.195 ± 0.122	1.091 ± 0.049		0.865 ± 0.038	0.872 ± 0.014
isoborneol	1.470 ± 0.112	1.310 ± 0.053	1.224 ± 0.045	1.076 ± 0.094	1.205 ± 0.051	x	0.971 ± 0.058
	1.408 ± 0.062	1.277 ± 0.056	1.192 ± 0.030	1.088 ± 0.087	1.175 ± 0.052		0.937 ± 0.052
fenchyl alcohol	1.423 ± 0.141	1.317 ± 0.043	1.348 ± 0.143	1.397 ± 0.081	1.244 ± 0.031	1.317 ± 0.079	x
	1.430 ± 0.143	1.290 ± 0.026	1.313 ± 0.128	1.373 ± 0.097	1.185 ± 0.019	1.272 ± 0.070	

**Table 3:** Study results obtained for Fe(II) chelation presented as EDTA equivalents for selected monoterpene concentrations (n = 4, mean values ± SD).

Monoterpene	Concentration [mM]	EDTA Equivalents [µg/mL]
*β*-Pinene	5	1.376 ± 0.002
	0.312	0.995 ± 0.031
Fenchone	5	1.076 ± 0.022
	0.312	0.912 ± 0.126
Verbenone	5	1.247 ± 0.037
	0.312	0.970 ± 0.011
Myrtenal	5	1.292 ± 0.023
	0.312	0.987 ± 0.044
Isoborneol	5	0.911 ± 0.004
	0.312	0.832 ± 0.020
Fenchyl alcohol	5	1.011 ± 0.005
	0.312	0.994 ± 0.018
3-Carene	5	1.361 ± 0.014
	0.312	1.341 ± 0.032

**Table 4:** CUPRAC study results presented in the form of EDTA equivalents [µg/mL] (n = 4, mean values ± SD).

Monoterpene	Concentration [mM]	EDTA Equivalents [µg/mL]
*β*-pinene	5	1.877 ± 0.075
	0.312	0.611 ± 0.015
Fenchone	5	0.809 ± 0.017
	0.312	0.796 ± 0.006
Verbenone	5	1.265 ± 0.026
	0.312	0.524 ± 0.006
Myrtenal	5	0.741 ± 0.019
	0.312	0.698 ± 0.020
Isoborneol	5	0.340 ± 0.006
	0.312	0.630 ± 0.022
Fenchyl alcohol	5	0.722 ± 0.014
	0.312	0.864 ± 0.014
3-Carene	5	3.062 ± 0.085
	0.312	0.654 ± 0.016

## Data Availability

The research data are available from the authors.
